# Diagnostic value of full-mouth radiography in horses

**DOI:** 10.3389/fvets.2022.971886

**Published:** 2022-10-05

**Authors:** Ian Tyler Bishop

**Affiliations:** Northern Equine Veterinary Services, Kirkfield, ON, Canada

**Keywords:** equine, horse, full-mouth, radiography, diagnostic, dentistry

## Abstract

In this observational study, oral examinations and full-mouth dental radiographs were performed on 248 horses presented for routine oral health care. The findings were assessed to determine how often disease was diagnosed by radiographs without having related findings on oral examination. In 50 horses (20%), there were radiographic signs of disease that would not have been predicted based upon the oral examination. 113 horses (46%) had oral examination findings that would have prompted dental radiography, according to the criteria of the study. Of these 113 horses, 24 (21%) had radiographic signs of disease that would have been missed if only targeted radiographs had been taken, rather than a full-mouth survey.

## Introduction

The objective of this study was to determine the value of performing full-mouth dental radiography for the diagnosis of dental and sinus disease in horses that were presented for routine oral care with no prior history of dental disease.

A full-mouth radiograph set provides diagnostic images of the complete dentition and supporting structures. Extraoral open-mouth and intraoral radiographic views for horses have been established and well described (1). In the equine patient, radiographic signs of apically infected cheek teeth include periapical alveolar bone sclerosis, root resorption (“clubbing” of tooth roots) and periapical alveolar bone lysis (“halo”). While computed tomography has been demonstrated to be more sensitive than radiographs in the diagnosis of dental pathology, this imaging modality is not readily available in most practices (2–4). Radiography, however, is typically available in equine veterinary practices.

Periodontal disease is assessed by a combination of oral examination (periodontal pocket depth measurements, bleeding indices) and radiographic estimation of attachment loss (5–7). Accurate and detailed examination of pockets can be challenging in the standing, sedated horse. Diastemata may be too narrow to allow insertion of a probe and even well-sedated horses often resist probing of painful periodontal pockets. Additionally, the reserve crown/root length of teeth is highly variable. These factors make radiographic assessment of attachment loss especially important in the horse. It is also worth noting that extraoral dental radiographs have limitations when assessing attachment loss. Oblique radiographic views may artifactually lengthen or foreshorten the teeth, and superimposition of structures obscures details and can make assessment of attachment loss challenging.

In the horse, the assessment of crown fractures for pulp exposure or near pulp exposure by oral examination can be challenging. Fractures are often irregular, may extend between tightly apposed teeth, and openings into pulp horns can be oblique and very small. The thickness of subocclusal secondary dentin may be as little as 2 mm over individual pulp horns (8). If there has been pulp exposure or near pulp exposure, oral examination alone will not reveal whether the pulp has responded with a reparative dentin bridge or if there has been pulp necrosis. While not sensitive in evaluating the internal structure of cheek teeth, radiographic imaging to assess for signs of apical changes is necessary to assess the vitality of a tooth.

If a tooth undergoes pulp necrosis due to anachoresis, dentin production stops and pulp horns will become exposed by normal dental wear. Initially, an opening into a necrotic pulp horn may appear small and innocuous but can immediately allow entrance of oral microflora and feed material into the pulp horn. Limited pulp necrosis occlusally with concurrent formation of a reparative dentin bridge may limit the occlusal defect to a shallow pit that will become inapparent over time with normal occlusal attrition. In some cases, a significant portion of occlusal pulp may necrose while reparative dentin is laid apically. In this case, the resulting secondary dentin defect may be relatively large and probe deeply but the tooth is still vital (9). The clinical finding of an occlusal defect over a pulp horn, while not diagnostic of a nonvital tooth, is an indication for radiographic assessment. Dental radiography does not have the sensitivity to detect dentinal bridging in premolars and molars; this evaluation requires a high resolution (1-2 mm slice thickness) CT scan.

The prevalence of cemental defects in infundibula is high and their shape is widely variable. Oral examination shows only the occlusal surface of the infundibulum and cannot predict the apical character of a defect. Only rough estimations of the severity of potential endodontic disease can be made based upon the staging of infundibular caries, their probing depth, and the patient's level of dental attrition or ipsilateral disease (10, 11). A grading system for staging infundibular caries has been described in the literature based upon the tissues affected. Grade 1: Caries of infundibular cementum only. Grade 2: Caries of infundibular cementum and enamel. Grade 3: Caries of infundibular cementum, enamel, and dentin. Grade 4: Caries of mesial and distal infundibula with coalescence. Grade 5: Infundibula associated dental fracture, apical disease, or tooth loss (11).

Other oral and paranasal sinus conditions that may be diagnosed radiographically but be inapparent on an oral exam include Equine Odontoclastic Tooth Resorption and Hypercementosis (EOTRH) (12–14), retained teeth, neoplasms and other masses or fluid in the paranasal sinuses, periapical masses, radicular cysts, root and reserve crown fractures, and dysplasia of the roots or reserve crown.

The diagnostic yield of survey dental radiographs has been established in dogs and cats and found to be high enough to justify the inclusion of full-mouth radiographs in the standard oral health assessment. Verstraete et al. (15, 16) found important findings on radiographs of teeth without clinical lesions at a rate of 28.7% in dogs and 41.7% in cats.

Recommendations by the American Dental Association and US Department of Health and Human Services for use of oral radiographs in humans depend upon the patient's age, history of dental disease, reported symptoms, and current clinical presentation (17). Targeted oral radiographs based upon the risk factors of each patient, or survey radiographs at 12–36 month intervals, are recommended.

Differences between human and equine dental practice should be considered when developing our own radiographic diagnostic protocols. Veterinary patients, unlike human patients, are unable to report symptoms that would prompt a human dentist to investigate. Also, the accumulated life-time radiation exposure is of limited concern for patients who do not live as long as humans and are not as regularly exposed. Conversely, equine veterinary staff are at a greater risk of radiation exposure than human or companion animal dental providers because the current technology and patient compliance require the operator to be patient-side rather than allowing them to leave the room.

The aim of this study was to aid in deciding when to recommend dental radiographs for equine patients by measuring how often disease is diagnosed on radiographs that would not have been predicted by oral exam.

## Materials and methods

Patients were presented on-farm for routine oral health assessment and occlusal adjustment to Ian Bishop, DVM during his 3rd and 4th year as an AVDC resident. Their name, age, sex and breed were recorded as given by the owner. In 60/248 cases, the age was not given and was estimated based upon dentition. Ages were estimated at <6 months, 1, 2, 3, 4, 5–9, 10–14, 15–19 or 20+ years of age based upon eruption times (18) and by comparing radiographic length of reserve crown to horses with known ages.

A brief physical exam was performed to screen for contraindications to standing sedation. The patient was sedated with detomidine (20–40 μg/kg IV) and butorphanol (0–0.01 mg/kg IV), moved into stocks, and given 5 min for sedation to take effect. After thoroughly rinsing the mouth, a detailed oral examination was performed with a bright headlight, full mouth speculum and mirror, and findings were recorded for each patient in the format of a 5-component exam (19).

1. Extraoral, assessing for symmetry, musculature, lymph nodes, swellings, odor, and discharge.
2. Oral Soft Tissue, noting abrasions, ulcerations, lacerations, and masses.
3. Occlusion, noting complement of teeth, position and orientation of teeth, length and contour of the clinical crowns, and relationship of the jaws.
4. Periodontal, noting gingivitis, gingival recession, periodontal pockets, diastemata, and mobile teeth.
5. Endodontic, noting dental fractures, enamel infractions, secondary dentin defects, peripheral caries and infundibular caries.

Staging and grading systems from the American Veterinary Dental College (AVDC) were used, such as those of periodontal disease, tooth resorption, infundibular caries, and mobility (20). The AVDC infundibular caries grading system varies slightly from the system discussed above: Grade 1-3 are identical while grade 4 is defined as “Affecting the structural integrity of the tooth” and there is no grade 5.

Regardless of oral exam findings, a set of dental radiographs was then taken using a Universal Imaging Stalo DR Cannon 810C plate system running CXDI Control Software NE v. 2.17.0.14 and Universal Imaging Ultra 90BT portable x-ray generator. The manufacturers recommended settings for sinus and dental radiographs of kVp 80 and mAs 1.0 were used. The dental radiograph set included the following views: Dorsoventral and lateral views of the sinuses, open-mouth extraoral dorsoventral oblique (30 degree) and ventrodorsal oblique (45 degree) views of the maxillary cheek teeth, open-mouth extraoral ventrodorsal oblique (45 degree) views of the mandibular cheek teeth and intraoral bisecting angle views of the incisors and canines (1). A single image was taken for maxillary incisors and mandibular incisors for mares and geldings, and for each canine in geldings. The beam angle was adjusted based upon the age and degree of dental attrition of the patient, in order to eliminate superimposition of left and right quadrants and minimize deformation of the image.

Findings that were indicative of periodontal, endodontic, or sinus disease from the oral examinations and radiographic reports were summarized in a spreadsheet for tabulation. Tables 1, 2 list the classes of abnormalities that were recorded. Anatomical/Occlusal abnormalities were included that may require treatment such as retained deciduous teeth requiring extraction and missing teeth which may be impacted, fractured below the gingiva or otherwise present and abnormal.

**Table 1:** Tabulated oral examination findings.

**Extraoral**	**Periodontal**	**Endodontic**	**Anatomical/Occlusal**
Nasal discharge	Diastemata	Crown fractures	Retained deciduous teeth
Draining tracts	Gingival recession	Secondary dentin defects	Missing teeth
Swellings	Periodontal pocket	Infundibular caries	
	Increased mobility	Parulis lesions	

**Table 2:** Tabulated radiographic findings.

**Paranasal sinuses**	**Periodontal**	**Endodontic**	**EOTRH**
Fluid lines	Attachment Loss	Fractures	Inflammatory tooth resorption
Abnormal opacities		Periapical sclerosis	Hypercementosis
		Periapical “halo”	
		Root resorption	
		Failure of the pulp chamber to narrow	
		Periapical opacities	
		Dysplasia of the reserve crown or roots	
		Infundibular dysplasia	
		Focal tooth resorption	
		Focal hypercementosis	

Each tabulated oral examination finding (Table 1), excluding infundibular caries Grade 1 and 2, was considered an indication to take dental radiographs.

The findings were listed in table format with the patients divided into age groups (Tables 3–**6**). A patient was counted once for each finding, regardless of how many teeth were affected. For example, a patient with multiple teeth with one or more secondary dentin defects would be counted once under “Patients with secondary dentin defect(s).” If they also had radiographic signs of apical infection associated with one or more of those teeth, they would have been counted once under “Patients with secondary dentin defect(s) and related radiographic signs of endodontic disease.” Patients with multiple unrelated findings would be counted once for each finding. For example, a horse with radiographic findings of EOTRH and periodontal disease of 308, 309 and 310 would be counted once for “Patients with signs of EOTRH” and once for “Patients with signs of periodontal disease.”

**Table 3:** General dental and sinus disease.

	** <5**	**5–9**	**10–14**	**15–19**	**20+**	**Total**
All patients	23 (9.3)	61 (24.6)	61 (24.6)	59 (23.8)	44 (17.7)	248
Patients with any signs of dental or sinus disease	7 (30.4)	30 (49.2)	36 (59.0)	38 (64.4)	35 (79.6)	146 (58.9)
Patients with oral exam findings that would prompt dental radiographs	4 (17.4)	25 (41.0)	29 (47.5)	25 (42.4)	30 (68.2)	113 (45.6)
Patients with radiographic signs of dental or sinus disease, regardless of oral exam findings	3 (13.0)	8 (13.1)	15 (24.6)	17 (28.8)	28 (63.6)	71 (28.6)
Patients with radiographic signs of dental or sinus disease without any exam findings that would prompt dental radiographs	2 (8.7)	2 (3.3)	3 (4.9)	8 (13.6)	4 (9.1)	19 (7.7)
If targeted radiograph had been taken based upon oral exam findings, other dental or sinus disease would have been missed *	0 (0)	1 (4)	3 (10.3)	9 (36.0)	11 (36.7)	24 (21.2)
Patients with radiographic signs of dental or sinus disease without related oral exam findings	2 (8.7)	4 (6.6)	7 (11.5)	16 (27.1)	21 (47.7)	50 (20.2)
Patients immediately above where treatment was recommended rather than monitoring or re-examination	0	0	2 (3.3)	7 (11.9)	4 (9.1)	13 (5.2)
Patients with radiographic signs of sinus disease without related oral exam findings	0	1 (1.6)	0	2 (3.4)	3 (6.8)	6 (2.4)
Patients with radiographic signs of endodontic disease without related oral exam findings (Other than EOTRH)	2 (8.7)	3 (4.9)	3 (4.9)	3 (5.1)	4 (9.1)	15 (6.0)

Percent in () for total number of horses in column.

^*^Given as percentage of “Patients with oral exam findings that would prompt dental radiographs”.

When measuring differences between age groups, the Chi-squared test for linear trend in proportions and two-sample z-test for difference in proportions were applied using Microsoft Office Excel 2016.

## Results

**Figure 1:**
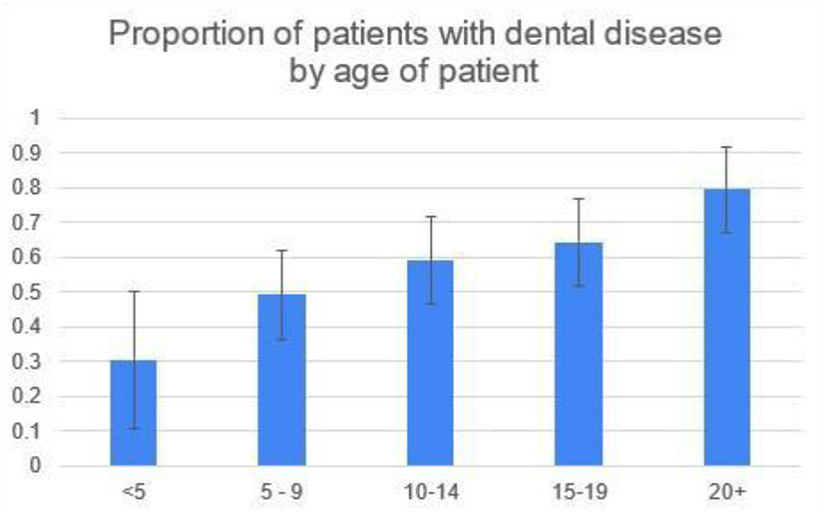
The percent of patients with dental disease increased with age, significant at the 5% level using a Chi-squared test for linear trend in proportions.

The number of patients in the age groups of 5–9, 10–14, and 15–19 were very close at 61, 61, and 59 respectively, while the 20+ group contained 44, and the <5 group had 23 horses (Table 3). There were a total of 248 patients included in the study. 146/248 (59%) of patients had signs of dental disease on oral examination and/or radiographs. The percent of patients with dental disease increased with age and this was significant at the 5% level using a Chi-squared test for linear trend in proportions (Figure 1).

**Figure 2:**
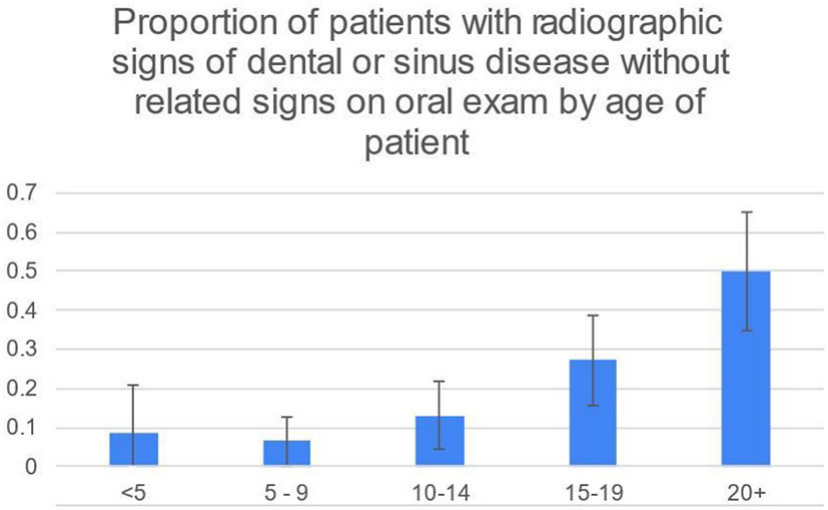
While the 95% CIs for 20+ and 15–19 overlap slightly, the difference in the percentage of 20+ and 15–19 age groups is significant at the 5% level using the two-sample z-test for difference in proportions.

50/248 (20%) of patients had radiographic signs of dental or sinus disease without related signs on oral examination. This was most common in the 20+ age group (21/44, 48%) and second most common in the 15–19 age group (16/59, 27%) (Figure 2). 19/248 (8%) patients had radiographic signs of disease without any oral examination findings that would prompt taking radiographs.

In 37/50 (74%) cases that had radiographic signs of dental or sinus disease without related signs on oral examination, the radiographic findings prompted the recommendation of a re-examination to confirm a diagnosis or track its progression, rather than immediate treatment (Table 3). This was based upon the clinical judgment of the author. For example, stage 2 tooth resorption (EOTRH) or the incidental finding of a sinus cyst in a senior patient prompted the recommendation of radiographic monitoring while finding a cheek tooth with periapical sclerosis, “halo” and root resorption would prompt recommendation of extraction. Of all patients, 13/248 (5%) had radiographic signs that resulted in immediate recommendation for treatment, in horses without related signs on oral examination. Of those, 12 were cases of EOTRH with stage 3 or greater tooth resorption in horses 10 years and older and 1 was a 20+ year old horse with periapical alveolar bone sclerosis and lysis of the 306.

**Figure 3:**
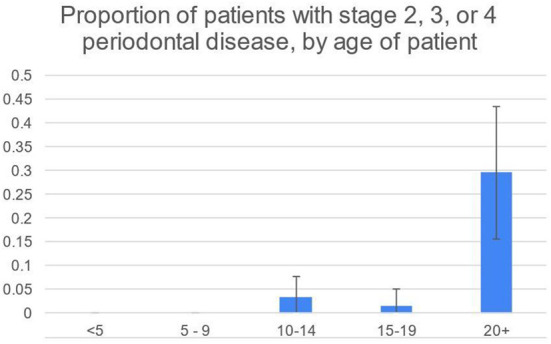
Proportion highest in 20+ group, according to the two-sample z-test for difference in proportions.

**Figure 4:**
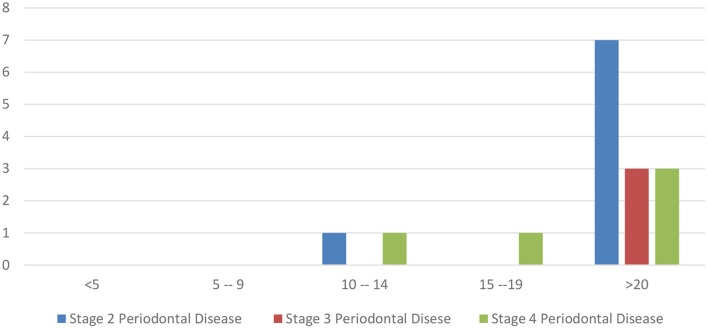
Number of horses with stage 2, 3 or 4 periodontal disease.

50/248 (20%) patients had some degree of diastemata, feed trapping and gingivitis between teeth (Table 4). Of those, 16/50 (32%) had attachment loss rather than only gingivitis. The prevalence of stage 2, 3 and 4 periodontal disease was highest in animals 20+ years old, according to the two-sample z-test for difference in proportions (Figures 3, 4).

**Table 4:** Periodontal disease.

	** <5**	**5–9**	**10–14**	**15–19**	**20+**	**Total**
Patients with signs of periodontal disease	1 (4.4)	7 (11.5)	15 (24.6)	7 (11.9)	20 (45.5)	50 (20.2)
Patients with no appreciable attachment loss	1 (4.4)	7 (11.5)	13 (21.2)	6 (10.2)	7 (15.9)	34 (13.7)
Patients with < 25% attachment loss	0	0	1 (1.6)	0	7 (15.9)	8 (3.2)
Patients with 25**–**50% attachment loss	0	0	0	0	3 (6.8)	3 (1.2)
Patients with >50% attachment loss	0	0	1 (1.6)	1 (1.7)	3 (6.8)	5 (2.0)

Percent in () for total number of horses in column.

6/16 (38%) patients with secondary dentin defects had associated radiographic signs of apical infection (Table 5). 7/44 (16%) patients with crown fractures were found to have signs of apical infection on radiographs. Radiographic signs of endodontic disease were not appreciated in cases with infundibular caries except when the caries was grade 4. However, the study population included only 1 case with up to grade 3 infundibular caries and only 2 cases of grade 4.

**Table 5:** Endodontic disease.

	** <5**	**5–9**	**10–14**	**15–19**	**20+**	**Total**
Patients with any oral exam findings of endodontic disease	2 (8.7)	14 (23.0)	19 (31.2)	28 (47.5)	16 (36.4)	79 (31.9)
Patients with secondary dentin defect(s)	1 (4.4)	5 (8.2)	4 (6.6)	2 (3.4)	4 (9.1)	16 (6.5)
Patients with secondary dentin defect(s) and related radiographic signs of endodontic disease	0	2 (3.3)	3 (4.9)	0	1 (2.3)	6 (2.4)
Patients with crown fracture(s)	1 (4.4)	7 (11.5)	11 (18.0)	15 (25.4)	10 (22.7)	44 (17.7)
Patients with crown fracture(s) and related radiographic signs of endodontic disease	0	0	5 (8.2)	1 (1.7)	1 (2.3)	7 (2.8)
Patients with infundibular caries	1	5	9	13	2	30 (12.1)
Patients with up to grade 1 infundibular caries	0	3	4	3	1	11 (4.4)
Patients with up to grade 2 infundibular caries	1	2	4	8	1	16 (6.5)
Patients with up to grade 3 infundibular caries	0	0	0	1	0	1 (0.4)
Patients with up to grade 4 infundibular caries	0	0	1	1	0	2 (0.8)
Patients with infundibular caries and related radiographic signs of endodontic disease	0	0	1	1	0	2 (0.8)

Percent in () for total number of horses in column.

EOTRH was the most common radiographic finding in horses without related signs on oral examination, with 29/248 (12%) cases. 28/103 (27%) patients 15 years or older had stage 2 or greater tooth resorption consistent with EOTRH (Table 6) and 25/28 (89%) of those patients had no signs on oral examination.

**Table 6:** Equine odontoclastic tooth resorption and hypercementosis.

	** <5**	**5–9**	**10–14**	**15–19**	**20+**	**Total**
Patients with radiographic and/or oral exam signs of EOTRH	0	0	4 (6.6)	12 (20.3)	16 (36.4)	32 (12.9)
Patients with only radiographic signs of EOTRH	0	0	4 (6.6)	11 (18.6)	14 (31.8)	29 (11.7)
Patients with stage 3 or greater tooth resorption	0	0	2 (3.3)	7 (11.9)	5 (11.4)	14 (5.7)
Patients with stage 3 or greater tooth resorption and only radiographic signs	0	0	2 (3.3)	6 (10.2)	3 (6.8)	11 (4.4)

113/248 (46%) horses had oral examination findings that would have prompted radiographs according to the criteria of this study. Of these cases, 24/113 (21%) had radiographic signs of disease in another part of the mouth without oral signs. These radiographic signs of disease would have been missed if only targeted radiographs had been taken, rather than a full-mouth survey.

## Discussion

This study examined the proportion of horses with radiographic signs of dental and sinus disease in the absence of related oral examination findings. The aim was to assess the value of survey full-mouth radiographs as part of the standard oral health assessment for horses.

A limitation of the study was found with respect to crown fractures. While compiling the data for this study it became clear that the author had been diagnosing fractures as involving the pulp or not by assessing oral examination and radiographic findings together in the field. Therefore, this data could not be used to assess how many fractures did not appear to involve the pulp on oral examination but were later determined to have apical disease on radiographs. With a total of 44 cases with crown fracture(s) and 7 with associated apical radiographic signs, it is unlikely that this data set was large enough to contribute to that question, regardless.

6/16 (38%) of cases with secondary dentin defects were found to have related radiographic signs of apical disease. As per the rationale discussed in the introduction, it is not expected that the character of the defects (their size, shape or measurable depth) would predict whether there was apical disease. In this study the number of secondary dentin defects per tooth was not tabulated but it should be noted that patterns of pulp exposure and fractures in apically infected teeth have been explored in other studies (9).

EOTRH in senior horses was the most common radiographic finding of dental disease without oral examination signs. Of patients 15 years or older, 28/103 (26%) were found to have at least early stages of EOTRH. This is less than found in other studies. One primary difference is likely at the level of sensitivity for stage 2 tooth resorption between clinicians. Henry et al. (13) found tooth resorption in 149/169 (88.2%) of horses (13) and Rerhl et al. (14) found a 94% prevalence of at least minor changes associated with EOTRH (14). Both were investigating specifically for the prevalence of tooth resorption and used older populations of horses. Even given the lower prevalence found in this study, in the author's opinion, the prevalence of EOTRH was great enough to justify radiographic screening in senior horses.

To estimate the diagnostic value of full-mouth radiographs in horses, the costs of time, client funds and risk of radiation exposure to the veterinary staff must be balanced against the benefits of potential information for the improvement of the horse's welfare. Given that 20% of all patients had radiographic signs of dental or sinus disease without related oral examination findings, dental survey radiographs in every patient could be justified. However, the cost-benefit ratio may be improved by selecting patients based upon age and/or limiting the number of views. Of those 50 cases with radiographic signs of disease without related oral examination findings, 29 (58%, 12% of all cases) had EOTRH and would have been captured by screening incisor/canine radiographs in patients 10 years and older. Of the remaining 21 cases, 15 were 10 years or older. Full-mouth survey radiographs of horses 10 years or older would have detected 44/50 (88%) of the cases where there were radiographic signs of disease in the absence of oral examination findings. Of the remaining six cases, 1 had a small, suspected sinus cyst, 2 had dysplasia of the reserve crown/roots that could lead to future dental disease, and 3 had radiographic changes of the pulp chamber or periapical alveolar bone that indicated endodontic disease. These 6 cases represent about 2% of the total study population.

Based upon the above information, veterinarians may consider recommending full-mouth radiographs for all horses, all horses 10 years or older, or when signs of disease are discovered on oral examination. Given that a significant portion of the cost of taking radiographs is spent in the set-up of the patient and equipment and given the risk of missing other disease if only targeted radiographs are taken, the author recommends performing a full-mouth survey whenever taking dental radiographs.

The horses included in this study had not been seen by the clinician before, had no known history of dental disease and had no known previous dental radiographs obtained. Assessing subsequent annual full-mouth radiograph sets for the rate of new findings was beyond the scope of this study but the question of how often to recommend survey radiographs merits further investigation. In humans, survey radiographs are recommended every 12–36 months (17). Annual oral examination and dental prophylaxis under general anesthesia is widely recommended for dogs and cats, and radiographs are recommended as part of the comprehensive oral health assessment. Given this, is seems reasonable to recommend survey radiographs of horses in the range of 12–36 months.

The results of this study demonstrate that the diagnostic yield of survey dental radiographs is high enough to justify the inclusion of full mouth radiographs in the standard oral health assessment in the horse. Variations across veterinary practices will inform whether veterinarians recommend survey radiographs on every patient or target older patients and selectively investigate oral examination findings.

## Data availability statement

The raw data supporting the conclusions of this article will be made available by the authors, without undue reservation.

## Ethics statement

Ethical review and approval was not required for the animal study because the horses in this study underwent clinical procedures that make up part of standard clinical practice and no procedures were performed that were not considered necessary or ethical. Written informed consent for participation was not obtained from the owners because the clients / owners of these horses gave consent for the procedures which were performed as part of clinical practice.

## Author contributions

The author confirms sole responsibility for the following study conception and design, data collection, analysis and interpretation of results, and manuscript.

## Conflict of interest

The author declares that the research was conducted in the absence of any commercial or financial relationships that could be construed as a potential conflict of interest. The reviewer RB declared a past co-authorship with the author to the handling editor.

## Publisher's note

All claims expressed in this article are solely those of the authors and do not necessarily represent those of their affiliated organizations, or those of the publisher, the editors and the reviewers. Any product that may be evaluated in this article, or claim that may be made by its manufacturer, is not guaranteed or endorsed by the publisher.
